# Astrochemical Pathways to Complex Organic and Prebiotic Molecules: Experimental Perspectives for In Situ Solid-State Studies

**DOI:** 10.3390/life11060568

**Published:** 2021-06-17

**Authors:** Daniele Fulvio, Alexey Potapov, Jiao He, Thomas Henning

**Affiliations:** 1 Istituto Nazionale di Astrofisica, Osservatorio Astronomico di Capodimonte, Salita Moiariello 16, 80131 Naples, Italy; 2 Max Planck Institute for Astronomy, Königstuhl 17, D-69117 Heidelberg, Germany; he@mpia.de (J.H.); henning@mpia.de (T.H.); 3 Laboratory Astrophysics Group of the Max Planck Institute for Astronomy at the Friedrich Schiller University Jena, Institute of Solid State Physics, Helmholtzweg 3, 07743 Jena, Germany; alexey.potapov@uni-jena.de

**Keywords:** complex organic molecules, astrobiology, astrochemistry, interstellar medium, molecular ices, solid state, protoplanetary disks, star forming regions, comets

## Abstract

A deep understanding of the origin of life requires the physical, chemical, and biological study of prebiotic systems and the comprehension of the mechanisms underlying their evolutionary steps. In this context, great attention is paid to the class of interstellar molecules known as “Complex Organic Molecules” (COMs), considered as possible precursors of prebiotic species. Although COMs have already been detected in different astrophysical environments (such as interstellar clouds, protostars, and protoplanetary disks) and in comets, the physical–chemical mechanisms underlying their formation are not yet fully understood. In this framework, a unique contribution comes from laboratory experiments specifically designed to mimic the conditions found in space. We present a review of experimental studies on the formation and evolution of COMs in the solid state, i.e., within ices of astrophysical interest, devoting special attention to the in situ detection and analysis techniques commonly used in laboratory astrochemistry. We discuss their main strengths and weaknesses and provide a perspective view on novel techniques, which may help in overcoming the current experimental challenges.

## 1. Introduction

Over the next decades, it is expected that one of the main challenges in science will be to gain a clear understanding on the processes and phenomena linked to the concept of life how we know it, its origin and early evolution. Key questions, which need to be answered, include: (i) How did life begin and evolve on Earth? (ii) What are the conditions for the origins of life? Does life exist elsewhere in the Universe? (iii) What is life’s future on Earth and beyond? Answering these and similar questions requires the physical, chemical, and biological study of prebiotic systems and a deep understanding of the principles governing their evolution into more complex systems and, finally, into living matter. Astrochemistry combines astronomy and chemistry. It directly studies the aforementioned issues, with a special focus on the so-called “Complex Organic Molecules” (COMs), a term referring to those astrophysically relevant organic molecules consisting of six or more atoms, many of which are considered possible precursors of prebiotic molecules, such as amino acids (in turn, precursors of proteins) and nucleobases (in turn, precursors of DNA and RNA). Although COMs are considered of paramount importance from an astrochemical and astrobiological point of view, the reader should keep in mind that they are only relatively “complex” when studied from a chemical and biological perspective [1,2].

**Figure 1:**
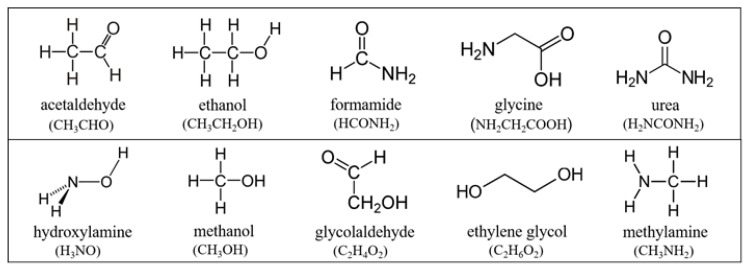
Structural formulas of some among the most astrophysically relevant COMs discussed in this review. The present figure was created by adapting original graphics taken from Wikipedia, the free encyclopedia (in particular, the graphic of acetaldehyde was created by UAwiki).

From the observational point of view, the main detection tool for molecules in space, in various astrophysical environments, is gas-phase radio astronomy supported by microwave, millimeter-wave, and terahertz spectroscopy. Complementary, solid-state materials are characterized by infrared (IR) spectroscopy. To date, more than 200 species have already been detected in the gas phase while only about 10 molecules have been spectroscopically identified in the solid state (e.g., Cologne Database for Molecular Spectroscopy and [3]). The variety, abundance, and distribution of gas-phase and solid-state COMs already detected or tentatively detected in space have been increasing in the last decades, touching various astronomical environments, from interstellar clouds, protostars, and protoplanetary disks (e.g., [1,2,3,4,5,6,7,8,9,10,11,12,13,14,15,16,17,18,19]) to the outer solar system, where COMs and prebiotic species have already been detected in several comets (e.g., [20,21,22,23,24,25,26,27,28,29,30,31]). To give the reader an idea, the list of these COMs includes but is not limited to: acetaldehyde (CH_3_CHO), ethanol (CH_3_CH_2_OH), formamide (HCONH_2_), glycine (NH_2_CH_2_COOH), and urea (H_2_NCONH_2_). Their structural formulas are shown in Figure 1. The detection of COMs in the solid state, i.e., in the icy component present in cold cosmic regions, such as dense molecular clouds of the interstellar medium (ISM), protostellar envelopes and protoplanetary disks (beyond the snowline), and on the surface of minor bodies of the solar system (comets, asteroids, satellites of planets, trans-Neptunian objects, …), is made difficult by the intrinsic problems related to IR spectroscopy. Simply said, the difficulties in identifying COMs in ices by IR spectroscopy are due to the unspecific nature of the signal coming from overlapping features belonging to different species having the same functional groups. Nevertheless, for solar system studies, space missions typically combine IR spectroscopy and mass spectrometry (MS). This allows the identification of COMs in situ (e.g., [27,28]), although MS carries its own intrinsic critical issues as well, such as the difficulty to distinguish molecules of the same mass and chiral isomers. A detailed discussion of these techniques can be found in the following sections of this review.

While most of the early astrochemistry models presumed that COM formation in the aforementioned astrophysical environments occurs through gas-phase reactions (e.g., [2,32,33,34]), the majority of current models assume that COM formation, especially the more complex ones, occurs mainly in the solid state, onto or within molecular ices found on cosmic dust grains and planetary surfaces. For the reader interested in understanding the nature and evolution of cosmic dust grains and their role in astrochemistry, we recommend a few review papers (e.g., [35,36,37,38,39,40,41,42]). Moving from earlier models to current ones has been favored by the comparison of theoretical simulations with new laboratory experiments and astronomical observations over the last few decades. Simply put, one of the main ideas beyond current astrochemistry models is that icy species may dissociate under energetic processing, such as UV irradiation and cosmic ray bombardment. Depending on the temperature of the ice, the so-formed fragments (radicals, ions,….) will have a certain mobility and, as a consequence, a certain efficiency/probability to re-combine. This way, new species may appear directly within the ice and, among them, COMs may be formed. As the ice eventually warms up, the mobility of the fragments increases and the efficiency/probability to re-combine and form new molecules increases as well. Finally, once the temperature is high enough for the ice to sublimate, it will enrich the gas-phase with the species it contains, including the more complex molecules (e.g., [1,15,43,44,45,46,47,48,49]). Alternative and complementary models have also been proposed, such as those based on reactions between stable molecules requiring external energy input by warming up the sample and on atom addition reactions. Additional details on the various mechanisms, triggering the formation and evolution of new molecules in ices found on cosmic dust grains and planetary surfaces, are briefly discussed in the following Section 2. However, a detailed discussion of these processes and mechanisms is beyond the scope of the current review and we refer the interested reader to dedicated papers/reviews ([49,50,51,52,53,54,55,56] and references therein).

The discussion so far indicates the importance of a comprehensive understanding of the physical–chemical mechanisms and processes underlying COM formation and evolution, especially in molecular ices. Most processes and mechanisms are not yet fully understood, and in this framework, laboratory studies specifically designed to simulate astrophysically relevant conditions play a unique role in improving our comprehension. Thanks to dedicated experiments, realistic scenarios towards molecular complexity can be established.

In this contribution, after a brief review of the main formation and evolution processes of COMs in space (Section 2), we focus on a key aspect of COM studies, often not comprehensively covered in reviews: the main strengths and weaknesses of in situ detection and analysis techniques commonly used in experimental astrochemistry (Section 3). The current experimental challenges and novel techniques to overcome present limitations will be the focus of Section 4.

## 2. Formation of COMs in the Solid State

Chemical processes leading to the formation of molecules in interstellar and circumstellar environments can be divided into two groups, gas-phase and solid-state surface reactions. Gas-phase formation routes to COMs are out of the scope of the present review and we refer the interested reader to a recent review paper on this topic [57]. In the following, we will focus on solid-state reactions.

In colder astrophysical environments, such as diffuse and dense clouds of the ISM, protostars, protoplanetary disks, and the outer solar system, gas-phase atoms and molecules may condense onto the cold surface of cosmic dust grains and planetary surfaces, thereby forming molecular ice. As an example, the ice films covering the micron-sized cosmic dust grains in dense molecular clouds are predominately made of H_2_O; the other main components being CO_2_ (up to about 35%), CO (up to about 30%), NH_3_ (below 5%), CH_4_ (below 5%), CH_3_OH (below 5%), H_2_CO (below 5%), OCN (below 2%), and OCS (below 2%). Values reported in parenthesis indicate abundances relative to H_2_O ice. The abundance values for each species may vary substantially for specific interstellar, circumstellar, or planetary environments (see, e.g., [13] and references therein). Starting from these species, larger molecules including COMs are formed in the ice by solid-state reactions. These are reactions triggered by energetic photon (UV, EUV, X-rays) irradiation, ion and electron bombardment, thermal processing, and atom addition. In dedicated review papers (e.g., [15,47,48]), the interested reader can find details on the experimental techniques and setups commonly employed in laboratory astrochemistry studies to recreate the different conditions present in astronomical sources. Within the laboratory astrochemistry community, irradiation by energetic photons, ions, and electrons is often referred to as “energetic processing”. In this case, energetic photons or particles impacting a solid-state target transfer energy into the system, causing the dissociation of stable molecules and leading to the formation of highly reactive radicals and ions. From these reactions, new species originally not present in the unprocessed ice can be formed.

By “thermal” reactions we typically mean reactions between stable molecules requiring additional external energy input by warming up the sample to overcome the reaction barrier. This energy input would depend on the specific solid-state system under study, ranging from a few dozen to hundreds of K. Atom addition reactions, typically studied at low temperatures (3–20 K), are often referred to in the literature as “non-energetic”; however, they have to also be considered as thermal reactions as atoms have a thermal distribution of speeds and are not excited by non-thermal processes. For these reactions, quantum tunneling may play an important role. There are a number of review papers devoted to the formation of COMs by the triggers mentioned above. We do not aim to duplicate them and, in the following, will refer the interested reader to these papers and will briefly discuss a few experimental examples of recent COM studies relevant to prebiotic chemistry. When possible, the importance of these COMs is also briefly put in the context of the direct comparison between laboratory results and astronomical observations.

### 2.1. Thermal Reactions

Atom addition reactions relevant to low-temperature astrophysical environments were reviewed by Linnartz et al. [53]. They discussed the pathways to simple and complex organic molecules of prebiotic interest, such as hydroxylamine (H_3_NO), methanol (CH_3_OH), and glycolaldehyde (C_2_H_4_O_2_). Their structural formulas are shown in Figure 1. Further laboratory experiments led to the conclusion that there may be no need for energetic processing to synthesize COMs in the solid state. For instance, Chuang et al. [58] showed that the hydrogenation (H-addition) of simple ices may result in the formation of glycolaldehyde (the smallest sugar), ethylene glycol (C_2_H_6_O_2_) (the simplest sugar alcohol), and methyl formate (an isomer of glycolaldehyde)—all these COMs are detected in astrophysical environments and in comets. Later, Ioppolo et al. [56] demonstrated that glycine (C_2_H_5_NO_2_) (the simplest amino acid) and its direct precursor methylamine (CH_3_NH_2_), both detected on comet 67P/Churyumov–Gerasimenko by the Rosetta mission [59], can be synthesized through atom and radical–radical addition surface reactions. Krim and Mencos [60] showed that N-addition to acetonitrile (CH_3_CN) (a potential amino acid precursor) leads to its chemical transformation into isomers CH_3_NC and CH_2_CNH with the abundance ratios explaining observations much better than the previous studies of such transformation induced by UV irradiation and particle bombardment. An alternative (to the hydrogenation of CO ice) route to methanol through H/O addition on the surface of laboratory analogues of cosmic carbonaceous grains was demonstrated by Potapov et al. [55]. Nguyen et al. [61] discussed the formation of amines (potential precursors of amino acids) through the hydrogenation of nitrile and isonitrile.

Thermal atom–molecule and molecule–molecule reactions as a step towards molecular complexity in astrophysical media were reviewed by Theulé et al. [49]. The authors classified molecules formed through thermal reactions according to their degree of complexity in an increasing order of generation. The largest molecules formed through molecule–molecule reactions initiated by warming up their samples (generation 2) included methylammonium methylcarbamate (CH_3_NH_3_^+^CH_3_NHCOO^-^) and alpha-aminoethanol (NH_2_CH(CH_3_)OH), both of which may act as amino acid precursors in astrophysical environments. The latter is a chiral molecule—a molecule with an important property when considering that important biological molecules have chirality as a feature. Amino acids in proteins are “left-handed” (L) and sugars in nucleic acids are “right-handed” (D) (see, e.g., [62]). One of the important open questions for laboratory astrochemistry is whether we can detect chirality in COM chemistry. Such detection would allow for a better understanding of the conditions responsible for the formation of prebiotic molecules and the astronomical search for chiral molecules. This will provide a link between molecules in space and life on Earth. Until now, only one chiral molecule, propylene oxide, has been detected in the ISM [63] in addition to amino acids detected in meteorites [64].

Following earlier investigations, recently, new studies devoted to the formation of ammonium carbamate (NH_4_^+^NH_2_COO^−^) through the CO_2_ + 2NH_3_ reaction were presented. Ammonium carbamate can be converted into urea (a possible precursor of pyrimidine required for the synthesis of nucleobases) and water, giving a start to a network of prebiotic reactions. It was shown that the reaction can be driven by structural changes evolving in amorphous water ice, such as pore collapse and crystallization, at high temperatures relevant to protostars and protoplanetary disks (i.e., above 100 K) [65]. Potapov et al. [66,67] demonstrated that the formation of ammonium carbamate on dust grains in astrophysical environments can be catalyzed by the surface of grains. Alpha-aminoethanol, ammonium carbamate and other COMs of prebiotic interest have not been detected in astrophysical environments because their gas-phase spectra are not available due to the instability of these molecules in the gas phase at room temperature. This highlights another important question—how do we measure gas-phase spectra of such COMs for their detection in astrophysical environments? Section 4.2 discusses one of the potential experimental possibilities.

### 2.2. Energetic Processing

In the well-known experiments of Miller and Urey, mimicking the possible conditions of the primitive Earth atmosphere, amino acids were formed starting from simple molecules, such as CH_4_, NH_3_, H_2_O, and H_2_, after exposing the gas mixture to an electrical discharge producing reactive radicals [68,69]. The idea of triggering reactions by energetically formed radicals has been later followed by a number of groups, leading to the production of amino acids and other prebiotic species on various gas-phase mixtures of simple molecules (e.g., [70,71,72] and references therein). This idea has also been applied to the extraterrestrial context, where astrochemically relevant ice mixtures are energetically processed, as discussed in the following.

Reactions triggered by energetic photons (UV, EUV, X-rays) and cosmic rays/solar wind (i.e., ions and electrons) bombardment fall into the group of energetic processing. Details on the possible mechanisms of COM formation induced by irradiation with energetic photons and cosmic ray/solar wind particles, relevant to low-temperature astrophysical environments, can be found in recent review papers (e.g., [15,38,47,48]).

We point out that experiments simulating the effects of the energetic processing of simple ices (e.g., H_2_O, CO, CO_2_) of astrophysical interest have been carried out in different astrochemistry laboratories around the world, already starting several decades ago. Discussing these studies in detail is beyond the scope of this review and we refer the interested reader to a few relevant papers: [73,74,75,76,77,78,79,80,81,82,83]. From the experimental expertise and data accumulated over the years, it is obvious that the increasing complexity of the starting ice mixture, UV irradiation and ion and electron bombardment experiments shows great potential in providing formation routes for COMs. Nevertheless, one should take into account that increasing the complexity of the starting ices leads to challenges in molecule identification by commonly used techniques (see Section 3 for a detailed discussion on this topic).

Focusing on some examples of ion and electron bombardment experiments, interesting results on possible pathways of amino acid formation were shown after the ion processing of ices of astrochemical relevance containing CH_3_CN or CH_3_CN:H_2_O [84]. Similarly, the formation of glycine was observed in processing experiments of ices, after ion irradiation of H_2_O:NH_3_:CO [85] as well as after electron irradiation of NH_3_:CH_3_COOD, CH_3_NH_2_:CO_2_ and CO_2_:CH_4_:NH_3_ mixtures ([86,87,88]). Another COM considered to have great potential in prebiotic chemistry—since it is the simplest molecule containing a peptide bond, known to be the “bridge” connecting amino acids in proteins and polypeptides—is formamide (NH_2_HCO). This species has been formed in several processing experiments of solid-state mixtures containing key astrophysical ices such as H_2_O, CO, CH_4_, NH_3_, HCN, or CH_3_OH, irradiated by energetic ions (e.g., [89,90]) and electrons (e.g., [91,92]). Its astrophysical importance is given by the fact that formamide has already been detected in several astrophysical environments, such as molecular clouds, protostars (e.g., [10,16]) and cometary comae (e.g., [20,25]). As an additional example, methyl formate (already discussed in Section 2.1) and other COMs were also observed after ion and electron bombardment of methanol-based ices (e.g., [47,93,94]).

Focusing now on photoprocessing experiments, it has been shown that the irradiation of planetary and interstellar ice mixtures by energetic photons (UV, EUV, X-rays) may also induce COM formation. For instance, different amino acids (some of which found in proteins), sugars and nucleobases were identified in the organic residues left over at room temperature after the irradiation of solid-state mixtures containing a combination of key astrophysical ices such as H_2_O, CH_3_OH, NH_3_, HCN, CO, and CO_2_ (e.g., [95,96,97,98,99,100,101]). The formation of simpler COMs, such as formamide and methyl formate, was also reported in photoprocessing experiments starting from different ice mixtures (e.g., [47,102,103,104,105,106]).

We point out that the unambiguous identification of COMs formed in situ during processing experiments of astrophysically relevant ices is often hampered by the used analyzing techniques, mainly infrared spectroscopy and mass spectrometry. The main problems related to these techniques, together with interesting novel techniques which may overcome their limitations, are the focus of Section 3 and Section 4.

## 3. Common Experimental Techniques Applied for In Situ Detection and Analysis of Solid-State COMs of Astrochemical Relevance

### 3.1. Infrared Spectroscopy

In the field of laboratory astrochemistry, Fourier transform infrared (FTIR) spectroscopy is one of the two main in situ experimental techniques, usually applied to detect molecules in the solid state. It uses a broadband thermal radiation source (e.g., globar, Hg lamp) and an interference pattern of a two-arm Michelson interferometer. In experiments with ices of astrophysical interest, typical resolutions, ranging from a few to 0.1 cm^−1^, are achieved by varying the interferometer path length. FTIR spectroscopy is used in one of the two main configurations, transmission or reflection mode. In both configurations, the radiation beam of the IR source points to the surface of the ice sample. When going through the ice, part of this radiation is absorbed at specific wavelengths which depend on the molecules constituting the sample (while the intensity of the absorption depends on the amount of absorbing molecules). The remaining part of the radiation goes through the ice and, when working in transmission mode, also through the substrate on top of which the sample was grown, while when in reflection mode, it is reflected by the substrate. For reflection spectroscopy, the term Fourier transform reflection absorption infrared spectroscopy (FT-RAIRS or, simply, RAIRS) is usually used. The IR beam can be at near normal incidence to the substrate, but FT-RAIRS mainly works at large angles of incidence with parallel-polarized light and on metal substrates because it takes advantage of the high electric field strength on the metal surface, which is only true for the field component normal to the surface, increasing the sensitivity of the technique. The major advantage of FT-RAIRS over transmission spectroscopy is that it can be used to detect intra-adsorbate and adsorbate–substrate vibrations and adsorbate–adsorbate interactions at monolayer and submonolayer coverages on metal surfaces [107]. On the other hand, a problem of using reflection spectroscopy is the presence of optical interference effects which often lead to erroneous measurements of absorption band strengths and give an apparent dependence of this quantity on film thickness, the index of refraction and wavelength [108].

FTIR spectroscopy allows for in situ and non-destructive characterization of the evolution of the composition and structure of ices before, during and after their thermal or non-thermal processing at defined experimental conditions, such as a specific temperature, starting ice composition, and thickness. Besides this, FTIR spectroscopy provides many additional possibilities, such as multiplex recording, broadband coverage and easy wavelength calibration. It provides reference spectra that can be used for interpreting astronomical IR spectra (however, mainly taken by space-borne observatories due to the absorption of IR radiation by the Earth’s atmosphere). FTIR spectroscopy is a very popular experimental technique worldwide. The reader can find many examples in the literature of experimental studies of COMs carried out by using FTIR spectroscopy (e.g., the review papers mentioned in the previous section and references therein).

**Figure 2:**
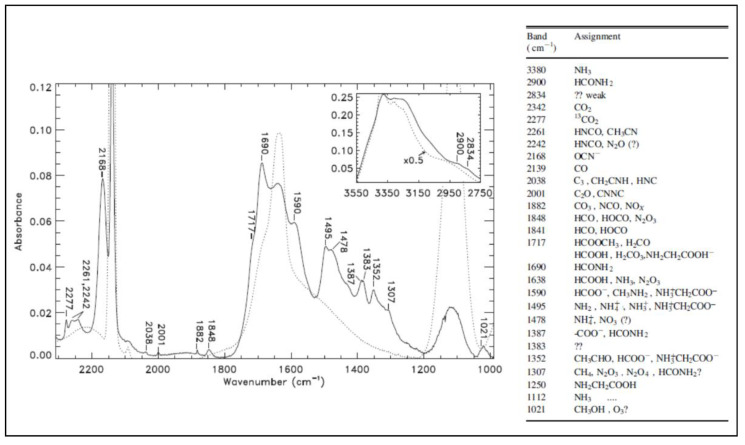
IR spectra of a H_2_O:CO:NH_3_ ice mixture (13 K) before (dotted line) and after (solid line) irradiation with soft X-ray for 2 h. In the figure, the upright inset panel shows the effects of photoprocessing in the range 2750–3550 cm^−1^. Numerical labels refer to the wavenumber of the main products of irradiation, identified in the table to the right. Adapted from [104], © AAS. Reproduced with permission.

However, the problem of IR spectroscopy as applied to the study of the formation and evolution of COMs in the solid state is that often several COMs show similar and overlapping broad spectral signatures belonging to different species, but having the same functional groups. An example is given in Figure 2, adapted from [104]. Moreover, an additional difficulty is typically related to the low efficiency of formation of new large and complex species within the ice, which leads to low amounts of COMs. In this case, the spectral features are very weak and the sensitivity of FTIR spectroscopy is not enough to detect them. Just to give the reader a reference value, the detection limit of FTIR spectrometers typically used in most laboratories is in the order of 0.1–1 monolayer of material, i.e., about 10^14^–10^15^ molecules cm^−2^ of the species under study. All this complicates the analysis and makes the identification of COMs difficult and limited. At smaller wavenumbers, in the terahertz and millimeter-wave spectral ranges, which are the ranges of many ground-based observatories (e.g., Effelsberg, GBT, NOEMA and ALMA), the signal to noise of FTIR spectrometers is limited due to the low power density of the thermal radiation source. Additionally, the discrete FT procedure becomes problematic at low frequencies because of the poor spectral resolution.

### 3.2. Mass Spectrometry

To acquire complementary information on the mass and composition of the “new” molecules formed in ices, solid-state FTIR spectroscopy is usually used in combination with gas-phase mass spectrometry (MS), the second key in situ experimental technique. In a typical MS experiment, species released from the solid state into the gas phase (process called desorption) reach the ionization region of a quadrupole mass spectrometer (QMS) by direct flight, are ionized and then ion signals for different m/z (mass to charge) ratios are detected by a mass spectrometer with a typical mass resolution m/∆m of several hundreds. MS allows one to follow molecular processes taking place within the ice before, during and after its processing by detecting and recording the species desorbed from the ice. As for FTIR spectroscopy, one can observe the evolution of ices at defined experimental conditions. MS has a very high sensitivity, capable of measuring partial pressures down to ~10^−13^ mbar [107]. MS in the laboratory allows for the direct comparison with in situ MS provided by solar system space missions such as Cassini−Huygens [109] and Rosetta [110]. The Rosetta mission has provided evidence for the presence of COMs and prebiotic species, e.g., glycine together with the precursor molecules methylamine and ethylamine, in cometary ices [27,59].

Mass spectrometry is usually applied to follow the kinetics of the desorption of molecules from ices in so-called temperature programmed desorption (TPD) experiments. In these experiments, after growing an ice sample with the molecules of interest (and its processing with one of the mechanisms discussed in Section 2), the ice is warmed up at a constant rate, inducing the desorption of molecules from its surface. Released molecules are detected in the gas phase by a mass spectrometer. Thus, one obtains information on the mass of a desorbed molecule and its desorption temperature and rate, which can be converted into a desorption energy. These quantities can then be used in astrochemical models. However, in the thermal desorption process, reactions may occur during the warm-up phase of the ice mixture. This makes it challenging to distinguish between COMs originally formed in the ice mixture at low temperature or during the heating process.

As for the case of FTIR, MS is a broadly used experimental technique. The reader can find many examples in studies on COM formation and evolution (see, e.g., the review papers mentioned in Section 2 and references therein).

**Figure 3:**
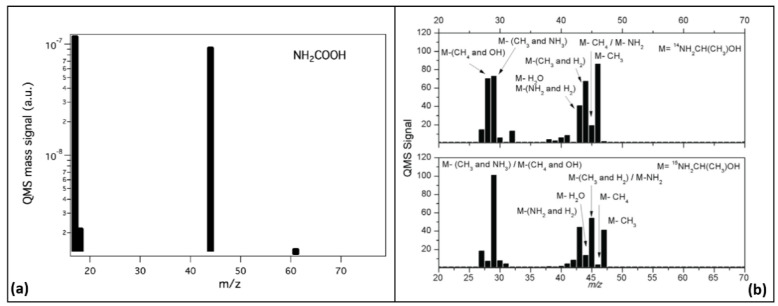
(**a**) Mass spectrum of NH_2_COOH at T = 265 K obtained using a quadrupole mass spectrometer. Republished with permission of Royal Society of Chemistry, from [111]; permission conveyed through Copyright Clearance Center, Inc. (**b**) The ^14^NH_2_CH(CH_3_)OH and ^15^NH_2_CH(CH_3_)OH mass spectra at 200 K. M is the mass of the molecular ion of NH_2_CH(CH_3_)OH m/z 61. Credit: adapted from [112], reproduced with permission © ESO.

As an example of a mass spectrum for COMs, we present in Figure 3 (left panel) the spectrum of carbamic acid (NH_2_COOH), recorded at 265 K [111]. Masses 17, 18, 44, and 61 correspond to ^14^NH_3_, ^15^NH_3_, CO_2_ and NH_2_COOH, respectively. This example highlights one of the main problems of a typical MS experiment. In the given example, the peak at m/z = 18 may be attributed to H_2_O instead of ^15^NH_3_ and therefore some of the molecules at m/z = 17 are OH molecules. The same problem of uncertain identification of the peak signals is clearly visible in the right panel of Figure 3 [112], where another drawback of MS is also shown: electron impact ionization of desorbed molecules leads to the fragmentation of the molecule and ambiguous attribution of the fragments. This is especially true for larger COMs, which are fragile and, under electron impact, can be easily destroyed. This implies that, by using MS, we do not detect the intact desorbed COM, but only its fragments. Moreover, MS suffers from several additional drawbacks which limit its use. Thermal desorption in a conventional TPD experiment is only able to desorb relatively volatile molecules (i.e., simple ones); for refractory molecules that only desorb above room temperature, alternative desorption methods are needed. In addition, conventional QMS has a rather limited sensitivity and mass resolution. Larger COMs produced in standard experiments usually have a relatively low production yield, often below the detection limit of the technique, implying that they cannot be detected by QMS. Finally, as the size of COM increases, it becomes increasingly difficult to identify the chemical formula of the molecule because of the low mass resolution. Consequently, it is not always possible to distinguish high mass COMs, molecules with similar masses, and structural and chiral isomers of the same molecule. The problem of high- and similar-mass COMs (but not the isomer problem) can be solved by using high-resolution mass spectrometers, such as time-of-flight (TOF) MS and Orbitrap, the latter reaching a mass resolution of 10^6^. However, specific structural isomers without their degradation (fragment-free) can be probed by using the tunable single photon vacuum ultraviolet photoionization in combination with reflectron time-of-flight mass spectrometry [113]. For details of these techniques, we refer the reader to Section 4.

### 3.3. Complementary Ex Situ Techniques

The main goal of in situ studies is to mimic physical–chemical processes taking place in space under particular conditions, such as low temperature and pressure, relevant to specific astrophysical environments. However, complimentary information can be obtained ex situ. Although outside the focus of this review, we want to mention some additional ex situ techniques used in complement to the techniques and methods discussed in the previous sections. As an example, two powerful ex situ methods to study COM produced by the processing of ices of astrophysical interest are gas chromatography–mass spectrometry (GC-MS) and high-performance liquid chromatography (HPLC). These methods were used, for instance, to identify prebiotic molecules produced by the energetic processing of ices such as ribose and other monosaccharides [99], amino acids [95,96] and nucleobases [114]. Other examples of relevant techniques are X-ray absorption near-edge structure (XANES) spectroscopy, energy-dispersive X-ray (EDX) spectroscopy, and transmission and scanning electron microscopy (TEM and SEM, respectively) used for the elemental analysis and chemical and structural characterization of samples. These and other ex situ methods are typically used when working with strongly bonded molecules, which pose a challenge to the thermal desorption technique. In such cases, after warming up the processed ices to room temperature, a refractory organic residue remains on the substrate. This residue can be constituted by COMs and prebiotic molecules, which cannot be fully characterized by conventional IR or MS techniques. After removing the residue from the vacuum chamber, it is ready to be analyzed by the aforementioned methods. However, the fact of working ex situ introduces additional problems, e.g., the opening for “undesirable” chemical reactions within samples during warming up and, after removing the residue from the vacuum chamber, with ambient air. This may alter the composition of the residue and affect, to some extent, the analysis results. A direct link to the astrophysical processes of interest taking place under low-temperature and low-pressure conditions can therefore be lost.

## 4. Experimental Challenges and Novel Techniques

In the previous section, we already discussed that infrared spectroscopy and mass spectrometry are the most widely used techniques in solid-state astrochemistry to characterize molecular ices and the desorption of molecules. Although important astrochemical pathways have been revealed by these techniques, their application is mostly limited to relatively simple molecules. As the size and number of COMs within the ice increase, it becomes more and more difficult to identify the exact molecules responsible for the observed IR features and MS signals. To overcome these difficulties and allow the identification of larger COMs, new techniques are being developed. Some of these techniques are discussed in this section.

### 4.1. Promising Mass Spectrometry Techniques

Molecules embedded in solid-state ices need to be desorbed into the gas phase for characterization by mass spectrometry. In addition to thermal desorption, laser desorption is a technique that gained popularity in chemical and biological studies (see, e.g., [115,116,117]) but started to be used in astrochemical studies only recently (e.g., [118,119]). Typically, a laser pulse, either in the UV or IR, is absorbed by a small column of the ice and generates a high intensity of heat in a relatively small spot size, increasing the temperature drastically. This leads to an instantaneous desorption of ice at the spot, releasing the molecules from the ice to the gas phase. As the desorption laser is usually pulsed, the technique works particularly well with mass spectrometers that rely on synchronization, such as time-of-flight mass spectrometers (TOF-MS). Although both UV and IR lasers are used for desorption, their performance varies with the specific system under study. UV lasers provide higher energy density than IR lasers, and may generate electronic vibrations of molecules. IR lasers are characterized by lower energy density and only excite the vibrational modes of molecules. For low absorption efficiencies of the molecules, the desorption yield would be relatively low. As a remedy to this problem, one could use an ice matrix that absorbs strongly at the laser wavelength. For this purpose, succinic acid, glycerol, urea, benzoic or cinnamic acid derivatives were used as matrices in prior studies [116,120,121]. However, in astrochemistry experiments, it makes sense to use an astrophysically relevant matrix. Water, being the main component of interstellar and planetary ices, is an ideal choice for the ice matrix. The possibility of using water ice as the matrix has already been demonstrated [119,122,123,124]. In these studies, an IR laser emitting at the frequency of the OH stretching mode is applied for efficient desorption. When designing laser desorption experiments, it is therefore important to choose the wavelength so that it overlaps with the infrared absorption peak of the ice matrix material. For this reason, one needs to know the wavelength dependence of the laser desorption yield for different matrix molecules. Such a study was carried out by Focsa et al. [125], who measured the laser desorption yield of water and ammonia. Similar measurements are desirable for other matrix candidates.

After desorption from the solid state, COMs need to be ionized before being detected by a mass spectrometer, which measures the mass to charge ratio. In QMS, molecules are typically ionized by electron impact. An electron energy of about 70–100 eV is usually chosen to have a balance between a high degree of ionization and a low degree of fragmentation. However, as the size of the molecule increases, the fragmentation probability increases. It is even possible that almost all the parent molecules are destroyed by electron impacts and one can only measure fragments. This makes the identification of the parent molecules a challenging task.

To solve the problems of destructive “hard” ionization by electron impacts, lasers have been employed as a source of less-destructive “soft” ionization. The energy of UV photons is much lower than the energy of 70–100 eV electrons and therefore UV light can ionize molecules without significant fragmentation [126]. When a UV laser is used, usually one desorbed molecule absorbs a photon, and is ionized to have a single charge with negligible fragmentation. The mass to charge ratio directly corresponds to the parent molecule. If a tunable UV laser is used, it is even possible to distinguish between molecules with the same mass or even the same chemical formula (i.e., isomers). This is because different molecules usually have different ionization energy thresholds. By selecting the photon energy to be above the threshold value, corresponding to one molecule while being below the value corresponding to the other molecule, one only ionizes the molecule with the lower ionization energy [113]. This provides extra information for the identification of COMs that is unavailable to conventional mass spectrometry. Resonance-enhanced multi-photon ionization (REMPI) is an alternative in situ method to ionize molecules (e.g., [127,128]). In REMPI experiments, molecules are first excited to an excited intermediate state by multiple photons, followed by ionization from the intermediate state by another photon or multiple photons. This technique has been applied by a number of groups to near ultraviolet spectroscopy of small molecules (H_2_, H_2_O) desorbed from ices of astrophysical interest in TPD experiments (e.g., [128,129]). However, so far, laser ionization has not gained wide application in the study of COM formation under conditions relevant to astrophysics. As the study of COM formation proceeds to larger and larger ones, laser ionization will have much to offer for the identification of larger COMs.

**Figure 4:**
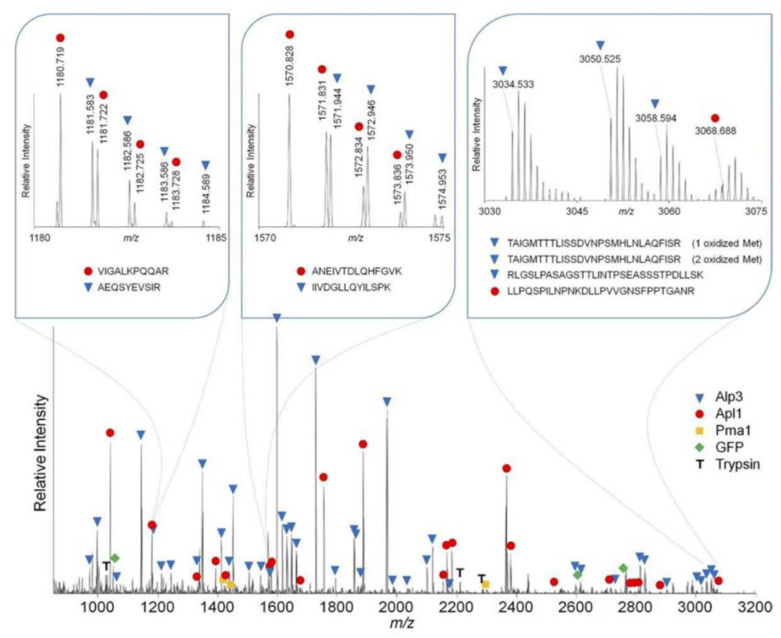
Representative MALDI LTQ Orbitrap MS of selected proteins. The panel insets show expanded MS regions containing Apl1 and Apl3 peptides. Adapted from [135], Copyright Elsevier (2010).

As discussed above, a laser is a powerful tool with tremendous potential for both the desorption and ionization of COMs. It is therefore natural to use a single laser for both aspects, thus exploiting their strength. Such a method, typically used in combination with a matrix, is called matrix-assisted laser desorption/ionization (MALDI). It has firstly gained popularity in chemistry and biomedicine after being introduced by Karas and co-workers [130]. Biological molecules as large as hundreds of thousands amu [122] are measured using MALDI without fragmentation. For a review of the application of MALDI to the identification of large biomolecules, we refer the reader to Clark et al. [131]. Figure 4 shows an example of the mass spectrum obtained from a MALDI experiment using a Linear Trap Quadrupole (LTQ) Orbitrap Mass Spectrometer as a detector. Here, a matrix which absorbs strongly at the wavelength of the laser is chosen. The intense heat generated by the laser pulse rapidly evaporates the matrix, forming a plume which contains the large biological molecules buried inside [121]. During the formation of the plume, the molecules are ionized. The exact mechanism of ionization is still debated, and several models are proposed as possible explanations of the ionization. For a detailed discussion, we refer the reader to dedicated reviews such as [132,133]. Both UV and IR lasers have been employed for MALDI. In the earlier years of MALDI, UV lasers were used almost exclusively [122]. Later on, since water is present in many biological samples and the IR beam absorbs strongly in the O–H stretching vibrational mode of water, there has been an increasing number of MALDI experiments using IR lasers. Niu et al. [134] compared the results of MALDI experiments performed by using IR and UV lasers, and showed that similar results were obtained from these two lasers. This suggests that the choice of the laser is largely dictated by the choice of the matrix material. In astrochemical studies of COM formation in astrophysically relevant ices, with water being the main component, the use of IR lasers would be a good choice.

**Figure 5:**
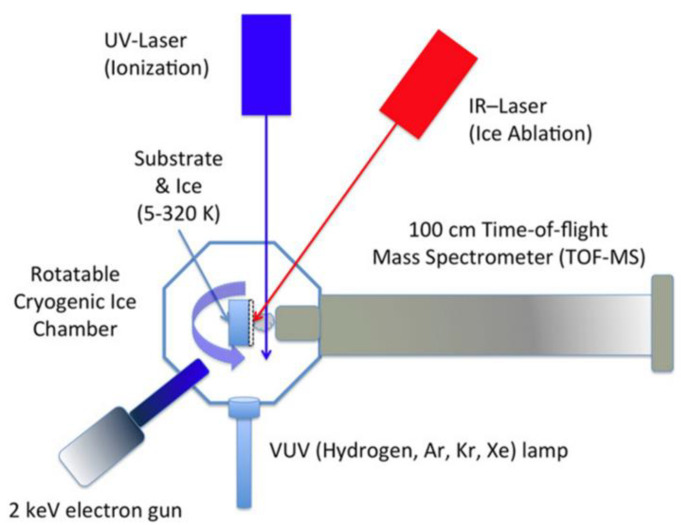
Schematic representation of the system developed at the Ice Spectroscopy Laboratory (ISL) at NASA Jet Propulsion Laboratory. The substrate is rotated for Vacuum Ultraviolet (VUV) irradiation and subsequent 2C-MALDI TOF-MS measurements, in situ, within the vacuum chamber. Adapted from [136], © AAS. Reproduced with permission.

Although MALDI is widely used in biological studies, it gained little attention in the astrochemical community. As far as we know, so far, there is only one astrochemistry laboratory that used the MALDI technique to identify COMs that are formed in astrophysically relevant ices [103,119,136]. The setup is shown in Figure 5. Gudipati and co-workers grew an ice mixture on a substrate kept at a temperature as low as 5 K, followed by irradiation with 2 keV electrons or UV photons, producing new species, including COMs such as formamide, acetamide, and methyl formate [103]. Subsequently, the ice mixture is exposed to a pulsed IR laser beam which desorbs and ionizes the molecules in the ice. To have a better ionization efficiency, an additional pulsed UV laser is also employed; therefore, they named the technique “two color” (2C) MALDI. It is synchronized with the IR laser but with a time delay. The delay is tuned so that the signal of the detected ions is optimized. The detector is a TOF-MS, by means of which COMs are identified. Gudipati and co-workers used one or more of the following three molecules as the matrix: water, ammonia and methanol. All these three molecules represent key species for astrophysical ices and have a relatively strong absorption in the IR, around 3 microns.

Ions produced by the ionization process, either with or without a matrix, are analyzed by a mass spectrometer to identify the parent molecules. Conventional QMS has a relatively low mass resolution and therefore is not ideal for the identification of larger COMs. High-resolution mass spectrometry (HRMS, resolution better than 10^4^) fills this gap and has been proven to be a powerful tool. A comprehensive review of HRMS can be found in [137]. There are three main types of HRMS: time-of-flight mass spectrometry (TOF-MS), Fourier transform ion cyclotron resonance mass spectrometry (FTICR-MS), and Orbitrap-MS. In TOF-MS, ions are accelerated by a high voltage, usually in the kV range, and travel in a flight tube. The time that the ions spent in the flight tube (i.e., time of flight) before reaching the detector depends on the mass to charge ratio of the ions. TOF-MS has a fast scan rate, but a lower sensitivity and lower mass resolution than the other two types of MS. Typically, high-resolution TOF-MS has a mass resolving power around 10^4^. Modern TOF-MS uses additional techniques, such as reflectron or multi-pass, to yield an even higher resolving power of a few 10^5^ ([137] and references therein). FTICR-MS and Orbitrap-MS are both Fourier transform mass analyzers. Both of them have a mass resolving power in the 10^5^–10^6^ range. FTICR-MS has a better performance in the lower mass range (less than a few hundred amu) [138]. In an FTICR-MS, a superconducting magnetic field confines the motion of ions to approximately cyclotron motion. The frequency of the cyclotron motion is related to the mass to charge ratio. The ions rotating at their resonance frequency induce an image current, which is measured and then Fourier transformed to the mass domain. Orbitrap-MS, instead of a superconducting magnet, uses an electrostatic field between a spindle-like central electrode and a barrel-like outer electrode for ion trapping. Due to the electrostatic force, ions cycle around the central electrode and simultaneously oscillate along its axis. Axial oscillations of ions are m/z-dependent and are detected by their image current produced on the outer electrode. Similar to FTICR-MS, this signal is measured and converted to the mass domain. Figure 4 shows an example of the mass spectrum obtained by an Orbitrap-MS. Since its introduction in 2004, Orbitrap-MS has seen a wide range of applications in the identification of large biomolecules. Due to its compact design of ion trapping, it is even being modified for future spaceflight missions [139]. For a more detailed discussion of Orbitrap-MS, we refer the interested reader to the reviews by [138,140].

To summarize, the combination of the aforementioned techniques, MALDI plus high-resolution mass spectrometry (TOF-MS, Orbitrap-MS, or FTICR-MS), shows great potential for the in situ detection and characterization of astrophysically relevant COMs. The final choice between the proper mass spectrometry technique to be coupled to MALDI would be defined by the resolution requirements of the specific system to be studied.

### 4.2. Promising Spectroscopy Techniques

As discussed in previous sections, even with a highly sensitive MS, it is not possible to identify structural and chiral isomers of COMs. However, there is an additional promising experimental approach to detect, in situ, COMs formed in solid-state or surface reactions and released into the gas phase. This possibility is provided by high-resolution gas-phase spectroscopy. High-resolution spectroscopy in the microwave (MW), millimeter-wave (MMW), terahertz (THz), and infrared (IR) spectral regions is a well-known and widely used tool to study rotational and ro-vibrational spectra of molecules and weakly bound molecular clusters. We refer the interested reader to the Handbook of high-resolution spectroscopy [141] and a couple of review papers [142,143]. High-resolution laboratory spectra are routinely used for the assignment of astronomical spectra obtained by ground-based radio telescopes.

High-resolution laboratory spectra provide unambiguous information on the molecular composition and structure of the analyzed sample because each molecule (including isomers) has its own, unique, spectroscopic signature due to the specific rotational and ro-vibrational energy levels defined by the composition and structure of the molecule. Importantly, it was shown that MW spectroscopy allows for the detection of molecular chirality [144,145]. Thus, high-resolution spectroscopy may also help in exploring the origin of the chirality of COMs.

**Figure 6:**
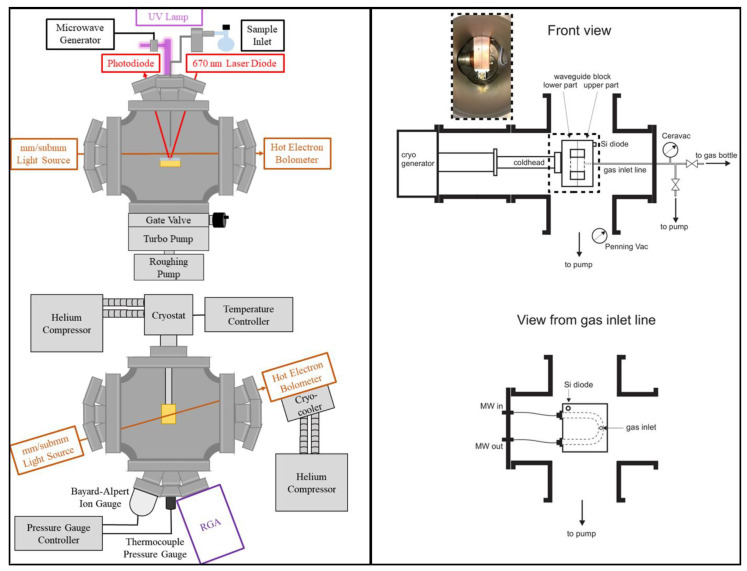
Left panel: Schematics of the side (upper left) and top view (lower left) of the experimental setup based on THz spectroscopy, adapted with permission from [146]. Copyright 2019, American Chemical Society. Right panel: Schematics of the front (upper right) and side view (lower right) of the experimental setup based on CP-FTMW spectroscopy, adapted with permission from [149]. Copyright 2020, American Chemical Society.

Combing desorption techniques discussed above with high-resolution gas-phase spectroscopy may potentially allow for the unambiguous identification and characterization of solid-state COMs. Indeed, this approach led to the independent development of two new experimental setups. Figure 6 shows the schematics of these setups.

One setup (shown in the left panel of Figure 6) is based on a combination of a broadband high-resolution THz spectrometer and a simple surface desorption experiment [146]. The setup allows for the direct absorption measurements of gas-phase spectra of desorbed species directly above the ice surface and is benchmarked on the detection of thermally desorbed H_2_O, D_2_O, and CH_3_OH. It was demonstrated that the detection limit of the technique is about 10^9^–10^10^ molecule cm^−3^, which is several orders of magnitude worse than the MS detection limit [107]. However, the sensitivity of such a setup can be improved by implementing multi-pass optics, providing many passes of the radiation through the sample and, thus, increasing the absorption path length. Examples of multi-pass optics in high-resolution spectroscopic studies can be found in the literature (e.g., ([147,148]). The setup also includes a 670 nm laser diode for monitoring the ice thickness and a UV lamp for inducing energetic processing of the pure ices.

The other setup (shown in the right panel of Figure 6) is a combination of a chirped pulse Fourier transform microwave (CP-FTMW) spectrometer and a U-shaped waveguide as a molecular cell mounted in a high-vacuum chamber, where this waveguide could be cooled to cryogenic temperatures [149]. The development of the CP-FTMW technique was performed by Pate and co-workers [150] and has quickly been used by several groups to obtain high-resolution gas phase spectra (e.g., [151,152,153,154]) and to study reaction dynamics (e.g., [155,156,157,158]). The ability of a CP-FTMW spectrometer to create a phase-reproducible chirped excitation pulse of more than 10 GHz linear frequency sweep and microsecond duration with subsequent broadband signal detection makes it possible to simultaneously detect many transitions, belonging to a single or several species with meaningful relative intensities, thus allowing one to follow the time evolution of spectra and reaction dynamics. In the experiments discussed in [149], ice samples were deposited onto the waveguide inner walls, and the desorbed molecules were detected inside the waveguide by using the CP-FTMW spectrometer with a 1 GHz frequency sweep. The setup was tested by measuring the high-resolution inversion spectrum of NH_3_ desorbed during the TPD experiments. The detection limit of the setup was estimated to be 6 × 10^−6^ mbar or 5 pmol of material. This is again several orders of magnitude worse than the MS detection limit; however, the setup has the advantage of providing broadband molecular spectra of desorbing volatiles. Potentially, the sensitivity of such a setup can be increased by increasing the power of the microwave excitation and decreasing the noise of the molecular response. However, the authors estimated that their setup should already be sensitive enough to study rotational spectra of COMs formed in the solid state and released into the gas phase. The authors also discussed possible modifications of the setup to perform UV-triggered surface chemistry.

To summarize, a new experimental approach—the combination of gas-phase high-resolution spectroscopy with a surface desorption technique—has been presented by two groups. Both experimental setups show potential for in situ COM detection and characterization. However, neither has been directly tested, so far, on COMs synthesized in surface reactions. For this, proof of the applicability and suitability of this new experimental approach is necessary.

## 5. Conclusions

Complex organic molecules (COMs) are considered to be potential precursors of prebiotic species, such as amino acids, sugars, and nucleobases. In this review, we discussed the nature of COMs and their detection and occurrence in several astrophysical environments such as interstellar clouds, protostars, protoplanetary disks, and the outer solar system. We provided the processes and mechanisms underlying the formation and evolution of COMs in the solid state, i.e., within molecular ices of astrophysical interest. We reviewed several fundamental experimental studies, which have been performed by different groups and with various setups and facilities, resulting in a great experimental effort and advancement toward the understanding of the COM formation mechanisms. Nevertheless, many questions are still open, such as those involving the identification and analysis of more complex COMs formed in situ during processing experiments of astrophysically relevant ices.

In this context, we discussed the main problems and limitations of infrared spectroscopy and mass spectrometry, the most commonly used experimental techniques for the study of solid-state COMs. Although these techniques have greatly contributed to the advancement of knowledge in the field of astrochemistry, their further contribution to this area of research has reached its intrinsic limit. The main problems of infrared spectroscopy and mass spectrometry are related to the identification and characterization of large molecules as the main scientific goals in the field of astrochemistry are moving towards species with increased molecular complexity. However, knowledge brought from other research fields (such as gas-phase spectroscopy, chemistry, and biology) provides the possibility to initiate novel experimental techniques in the field of solid-state astrochemistry, which show great potential to overcome the main experimental limitations of present experiments. Examples have been provided in this review, focusing on important technical details and demonstrating the advantages and benefits of these novel techniques.

We believe that the experimental route described in the present review will be the next big step for laboratory astrochemistry towards a better understanding of the astrochemical pathways from simple molecules to complex organic and prebiotic molecules.

## References

[1] Herbst E., van Dishoeck E.F. (2009). Complex Organic Interstellar Molecules. Annu. Rev. Astron. Astrophys..

[2] Jørgensen J.K., Belloche A., Garrod R.T. (2020). Astrochemistry during the Formation of Stars. Annu. Rev. Astron. Astrophys..

[3] McGuire B.A. (2018). 2018 Census of Interstellar, Circumstellar, Extragalactic, Protoplanetary Disk, and Exoplanetary Molecules. Astrophys. J. Suppl. Ser..

[4] Gibb E.L., Whittet D.C.B., Boogert A.C.A., Tielens A.G.G.M. (2004). Interstellar ice: The Infrared Space Observatory legacy. Astrophys. J. Suppl. Ser..

[5] Raunier S., Chiavassa T., Duvernay F., Borget F., Aycard J.P., Dartois E., d’Hendecourt L. (2004). Tentative identification of urea and formamide in ISO-SWS infrared spectra of interstellar ices. Astron. Astrophys..

[6] Arce H.G., Santiago-García J., Jørgensen J.K., Tafalla M., Bachiller R. (2008). Complex Molecules in the L1157 Molecular Outflow. Astrophys. J..

[7] Oberg K.I., Boogert A.C.A., Pontoppidan K.M., van den Broek S., van Dishoeck E.F., Bottinelli S., Blake G.A., Evans N.J. (2011). The Spitzer Ice Legacy: Ice Evolution from Cores to Protostars. Astrophys. J..

[8] Oberg K.I., Bottinelli S., Jorgensen J.K., van Dishoeck E.F. (2010). A Cold Complex Chemistry toward the Low-Mass Protostar B1-b: Evidence for Complex Molecule Production in Ices. Astrophys. J..

[9] Bacmann A., Taquet V., Faure A., Kahane C., Ceccarelli C. (2012). Detection of complex organic molecules in a prestellar core: A new challenge for astrochemical models. Astron. Astrophys..

[10] Kahane C., Ceccarelli C., Faure A., Caux E. (2013). Detection of Formamide, the Simplest but Crucial Amide, in a Solar-Type Protostar. Astrophys. J. Lett..

[11] Remijan A.J., Snyder L.E., McGuire B.A., Kuo H.L., Looney L.W., Friedel D.N., Golubiatnikov G.Y., Lovas F.J., Ilyushin V.V., Alekseev E.A. (2014). Observational Results of a Multi-Telescope Campaign in Search of Interstellar Urea [(Nh_2_)_2_Co]. Astrophys. J..

[12] Fayolle E.C., Oberg K.I., Garrod R.T., van Dishoeck E.F., Bisschop S.E. (2015). Complex organic molecules in organic-poor massive young stellar objects. Astron. Astrophys..

[13] Boogert A.C.A., Gerakines P.A., Whittet D.C.B. (2015). Observations of the Icy Universe. Annu. Rev. Astron. Astrophys..

[14] Lopez-Sepulcre A., Jaber A.A., Mendoza E., Lefloch B., Ceccarelli C., Vastel C., Bachiller R., Cernicharo J., Codella C., Kahane C. (2015). Shedding light on the formation of the pre-biotic molecule formamide with ASAI. Mon. Not. R. Astron. Soc..

[15] Oberg K.I. (2016). Photochemistry and Astrochemistry: Photochemical Pathways to Interstellar Complex Organic Molecules. Chem. Rev..

[16] López-Sepulcre A., Balucani N., Ceccarelli C., Codella C., Dulieu F., Theulé P. (2019). Interstellar Formamide (NH_2_CHO), a Key Prebiotic Precursor. ACS Earth Space Chem..

[17] Thiel V., Belloche A., Menten K.M., Giannetti A., Wiesemeyer H., Winkel B., Gratier P., Müller H.S.P., Colombo D., Garrod R.T. (2019). Small-scale physical and chemical structure of diffuse and translucent molecular clouds along the line of sight to Sgr B2. Astron. Astrophys..

[18] Bergner J.B., Martin-Domenech R., Oberg K.I., Jorgensen J.K., Artur de la Villarmois E., Brinch C. (2019). Organic Complexity in Protostellar Disk Candidates. ACS Earth Space Chem..

[19] Kleiner I. (2019). Spectroscopy of Interstellar Internal Rotors: An Important Tool for Investigating Interstellar Chemistry. ACS Earth Space Chem..

[20] Bockelee-Morvan D., Lis D.C., Wink J.E., Despois D., Crovisier J., Bachiller R., Benford D.J., Biver N., Colom P., Davies J.K. (2000). New molecules found in comet C/1995 O1 (Hale-Bopp)—Investigating the link between cometary and interstellar material. Astron. Astrophys..

[21] Elsila J.E., Glavin D.P., Dworkin J.P. (2009). Cometary glycine detected in samples returned by Stardust. Meteorit. Planet. Sci..

[22] Mumma M.J., Charnley S.B. (2011). The Chemical Composition of Comets-Emerging Taxonomies and Natal Heritage. Annu. Rev. Astron. Astrophys..

[23] Biver N., Bockelée-Morvan D., Moreno R. (2015). Ethyl alcohol and sugar in comet C/2014 Q2 (Lovejoy). Sci. Adv..

[24] Biver N., Bockelee-Morvan D., Debout V., Crovisier J., Boissier J., Lis D.C., Dello Russo N., Moreno R., Colom P., Paubert G. (2014). Complex organic molecules in comets C/2012 F6 (Lemmon) and C/2013 R1 (Lovejoy): Detection of ethylene glycol and formamide. Astron. Astrophys..

[25] Goesmann F., Rosenbauer H., Bredehöft J.H., Cabane M., Ehrenfreund P., Gautier T., Giri C., Krüger H., Le Roy L., MacDermott A.J. (2015). Organic compounds on comet 67P/Churyumov-Gerasimenko revealed by COSAC mass spectrometry. Science.

[26] Le Roy L., Altwegg K., Balsiger H., Berthelier J.-J., Bieler A., Briois C., Calmonte U., Combi M.R., De Keyser J., Dhooghe F. (2015). Inventory of the volatiles on comet 67P/Churyumov-Gerasimenko from Rosetta/ROSINA. Astron. Astrophys..

[27] Altwegg K., Balsiger H., Berthelier J.J., Bieler A., Calmonte U., Fuselier S.A., Goesmann F., Gasc S., Gombosi T.I., Le Roy L. (2017). Organics in comet 67P-a first comparative analysis of mass spectra from ROSINA-DFMS, COSAC and Ptolemy. Mon. Not. R. Astron. Soc..

[28] Altwegg K., Balsiger H., Fuselier S.A. (2019). Cometary Chemistry and the Origin of Icy Solar System Bodies: The View after Rosetta. Annu. Rev. Astron. Astrophys..

[29] Hadraoui K., Cottin H., Ivanovski S.L., Zapf P., Altwegg K., Benilan Y., Biver N., Della Corte V., Fray N., Lasue J. (2019). Distributed glycine in comet 67P/Churyumov-Gerasimenko. Astron. Astrophys..

[30] Schuhmann M., Altwegg K., Balsiger H., Berthelier J.J., De Keyser J., Fiethe B., Fuselier S.A., Gasc S., Gombosi T.I., Hänni N. (2019). Aliphatic and aromatic hydrocarbons in comet 67P/Churyumov-Gerasimenko seen by ROSINA. Astron. Astrophys..

[31] Rubin M., Bekaert D.V., Broadley M.W., Drozdovskaya M.N., Wampfler S.F. (2019). Volatile Species in Comet 67P/Churyumov-Gerasimenko: Investigating the Link from the ISM to the Terrestrial Planets. ACS Earth Space Chem..

[32] Charnley S.B. (1995). Hot core chemistry. Astrophys. Space Sci..

[33] Geppert W.D., Hamberg M., Thomas R.D., Osterdahl F., Hellberg F., Zhaunerchyk V., Ehlerding A., Millar T., Roberts H., Semaniak J. (2006). Dissociative Recombination of Protonated Methanol. Faraday Discuss..

[34] Garrod R.T., Herbst E. (2006). Formation of methyl formate and other organic species in the warm-up phase of hot molecular cores. Astron. Astrophys..

[35] Henning T., Salama F. (1998). Carbon in the Universe. Science.

[36] Draine B.T. (2003). Interstellar dust grains. Annu. Rev. Astron. Astrophys..

[37] Apai D., Lauretta D.S., Lauretta D.S., Apai D. (2010). Planet formation and protoplanetary dust. Protoplanetary Dust: Astrophysical and Cosmochemical Perspectives.

[38] Muñoz Caro G.M., Dartois E. (2013). Prebiotic chemistry in icy grain mantles in space. An experimental and observational approach. Chem. Soc. Rev..

[39] Jäger C., Gail H.-P., Rietmeijer F.J.M., Nuth J.A., Mutschke H., Mennella V. (2014). Formation of Nanoparticles and Solids. Lab. Astrochem..

[40] Jones A.P., Ysard N., Köhler M., Fanciullo L., Bocchio M., Micelotta E., Verstraete L., Guillet V. (2014). The cycling of carbon into and out of dust. Faraday Discuss..

[41] Potapov A., McCoustra M.R.S. (2021). Physics and chemistry on the surface of cosmic dust grains: A laboratory view. Int. Rev. Phys. Chem..

[42] Henning T. (2010). Cosmic Silicates. Annu. Rev. Astron. Astrophys..

[43] Garrod R.T., Widicus Weaver S.L., Herbst E. (2008). Complex chemistry in star-forming regions: An expanded gas-grain warm-up chemical model. Astrophys. J..

[44] Garrod R.T. (2013). A Three-Phase Chemical Model of Hot Cores: The Formation of Glycine. Astrophys. J..

[45] Cazaux S., Tielens A.G.G.M., Ceccarelli C., Castets A., Wakelam V., Caux E., Parise B., Teyssier D. (2003). The hot core around the low-mass protostar IRAS 16293-2422: Scoundrels rule!. Astrophys. J..

[46] Jørgensen J.K., Bourke T.L., Nguyen Luong Q., Takakuwa S. (2011). Arcsecond resolution images of the chemical structure of the low-mass protostar IRAS 16293-2422. Astron. Astrophys..

[47] Arumainayagam C.R., Garrod R.T., Boyer M.C., Hay A.K., Bao S.T., Campbell J.S., Wang J.Q., Nowak C.M., Arumainayagam M.R., Hodge P.J. (2019). Extraterrestrial prebiotic molecules: Photochemistry vs. radiation chemistry of interstellar ices. Chem. Soc. Rev..

[48] Sandford S.A., Nuevo M., Bera P.P., Lee T.J. (2020). Prebiotic Astrochemistry and the Formation of Molecules of Astrobiological Interest in Interstellar Clouds and Protostellar Disks. Chem. Rev..

[49] Theule P., Duvernay F., Danger G., Borget F., Bossa J.B., Vinogradoff V., Mispelaer F., Chiavassa T. (2013). Thermal reactions in interstellar ice: A step towards molecular complexity in the interstellar medium. Adv. Space Res..

[50] Vidali G. (2013). H-2 Formation on Interstellar Grains. Chem. Rev..

[51] Balucani N., Ceccarelli C., Taquet V. (2015). Formation of complex organic molecules in cold objects: The role of gas-phase reactions. Mon. Not. R. Astron. Soc..

[52] Fedoseev G., Cuppen H.M., Ioppolo S., Lamberts T., Linnartz H. (2015). Experimental evidence for glycolaldehyde and ethylene glycol formation by surface hydrogenation of CO molecules under dense molecular cloud conditions. Mon. Not. R. Astron. Soc..

[53] Linnartz H., Ioppolo S., Fedoseev G. (2015). Atom addition reactions in interstellar ice analogues. Int. Rev. Phys. Chem..

[54] Herbst E. (2017). The synthesis of large interstellar molecules. Int. Rev. Phys. Chem..

[55] Potapov A., Jäger C., Henning T., Jonusas M., Krim L. (2017). The Formation of Formaldehyde on Interstellar Carbonaceous Grain Analogs by O/H Atom Addition. Astrophys. J..

[56] Ioppolo S., Fedoseev G., Chuang K.J., Cuppen H.M., Clements A.R., Jin M., Garrod R.T., Qasim D., Kofman V., van Dishoeck E.F. (2020). A non-energetic mechanism for glycine formation in the interstellar medium. Nat. Astron..

[57] Cooke I.R., Sims I.R. (2019). Experimental Studies of Gas-Phase Reactivity in Relation to Complex Organic Molecules in Star-Forming Regions. ACS Earth Space Chem..

[58] Chuang K.-J., Fedoseev G., Qasim D., Ioppolo S., van Dishoeck E.F., Linnartz H. (2017). Production of complex organic molecules: H-atom addition versus UV irradiation. Mon. Not. R. Astron. Soc..

[59] Altwegg K., Balsiger H., Bar-Nun A., Berthelier J.J., Bieler A., Bochsler P., Briois C., Calmonte U., Combi M.R., Cottin H. (2016). Prebiotic chemicals-amino acid and phosphorus-in the coma of comet 67P/Churyumov-Gerasimenko. Sci. Adv..

[60] Krim L., Mencos A. (2019). Determination of [CH3NC]/[H2C=C=NH] Abundance Ratios from N + CH3CN Solid Phase Reaction in the Temperature Range from 10 to 40 K: Application to the Complex Chemistry in Star-Forming Regions. ACS Earth Space Chem..

[61] Nguyen T., Fourre I., Favre C., Barois C., Congiu E., Baouche S., Guillemin J.C., Ellinger Y., Dulieu F. (2019). Formation of amines: Hydrogenation of nitrile and isonitrile as selective routes in the interstellar medium. Astron. Astrophys..

[62] Bonner W.A. (1991). The Origin and Amplification of Biomolecular Chirality. Orig. Life Evol. Biosph..

[63] McGuire B.A., Carroll P.B., Loomis R.A., Finneran I.A., Jewell P.R., Remijan A.J., Blake G.A. (2016). Discovery of the interstellar chiral molecule propylene oxide (CH_3_CHCH_2_O). Science.

[64] Evans A.C., Meinert C., Giri C., Goesmann F., Meierhenrich U.J. (2012). Chirality, photochemistry and the detection of amino acids in interstellar ice analogues and comets. Chem. Soc. Rev..

[65] Ghesquiere P., Ivlev A., Noble J.A., Theule P. (2018). Reactivity in interstellar ice analogs: Role of the structural evolution. Astron. Astrophys..

[66] Potapov A., Jäger C., Henning T. (2020). Thermal formation of ammonium carbamate on the surface of laboratory analogs of carbonaceous grains in protostellar envelopes and planet-forming disks. Astrophys. J..

[67] Potapov A., Theule P., Jäger C., Henning T. (2019). Evidence of surface catalytic effect on cosmic dust grain analogues: The ammonia and carbon dioxide surface reaction. Astrophys. J. Lett..

[68] Miller S.L. (1953). A Production of Amino Acids under Possible Primitive Earth Conditions. Science.

[69] Miller S.L., Urey H.C. (1959). Organic Compound Synthesis on the Primitive Earth. Science.

[70] Parker E.T., Cleaves H.J., Callahan M.P., Dworkin J.P., Glavin D.P., Lazcano A., Bada J.L. (2011). Enhanced Synthesis of Alkyl Amino Acids in Miller’s 1958 H2S Experiment. Orig. Life Evol. Biosph..

[71] Wollrab E., Scherer S., Aubriet F., Carre V., Carlomagno T., Codutti L., Ott A. (2016). Chemical Analysis of a “Miller-Type” Complex Prebiotic Broth Part I: Chemical Diversity, Oxygen and Nitrogen Based Polymers. Orig. Life Evol. Biosph..

[72] Scherer S., Wollrab E., Codutti L., Carlomagno T., da Costa S.G., Volkmer A., Bronja A., Schmitz O.J., Ott A. (2017). Chemical Analysis of a “Miller-Type” Complex Prebiotic Broth. Orig. Life Evol. Biosph..

[73] Moore M.H., Donn B., Khanna R., A’Hearn M.F. (1983). Studies of proton-irradiated cometary-type ice mixtures. Icarus.

[74] Strazzulla G., Palumbo M.E. (1998). Evolution of icy surfaces: An experimental approach. Planet. Space Sci..

[75] Gerakines P., Moore M., Hudson R. (2001). Energetic Processing of Laboratory Ice Analogs: UV Photolysis versus Ion Bombardment. J. Geophys. Res. Planets.

[76] Hudson R.L., Moore M.H. (2002). The N-3 radical as a discriminator between ion-irradiated and UV-photolyzed astronomical ices. Astrophys. J..

[77] Huels M.A., Parenteau L., Bass A.D., Sanche L. (2008). Small steps on the slippery road to life: Molecular synthesis in astrophysical ices initiated by low energy electron impact. Int. J. Mass Spectrom..

[78] Muñoz Caro G.M., Dartois E., Boduch P., Rothard H., Domaracka A., Jimenez-Escobar A. (2014). Comparison of UV and high-energy ion irradiation of methanol:ammonia ice. Astron. Astrophys..

[79] Kim Y.S., Kaiser R.I. (2012). Electron irradiation of Kuiper Belt surface ices: Ternary N_2_–CH_4_–CO mixtures as a case study. Astrophys. J..

[80] Materese C.K., Cruikshank D.P., Sandford S.A., Imanaka H., Nuevo M. (2015). Ice chemistry on outer solar system bodies: Electron radiolysis of N_2_-, CH_4_-, and CO-containing ices. Astrophys. J..

[81] Boyer M.C., Rivas N., Tran A.A., Verish C.A., Arumainayagam C.R. (2016). The role of low-energy (≤20 eV) electrons in astrochemistry. Surf. Sci..

[82] Rothard H., Domaracka A., Boduch P., Palumbo M.E., Strazzulla G., da Silveira E.F., Dartois E. (2017). Modification of ices by cosmic rays and solar wind. J. Phys. B At. Mol. Opt. Phys..

[83] Ada Bibang P.C.J., Agnihotri A.N., Augé B., Boduch P., Desfrançois C., Domaracka A., Lecomte F., Manil B., Martinez R., Muniz G.S.V. (2019). Ion radiation in icy space environments: Synthesis and radioresistance of complex organic molecules. Low Temp. Phys..

[84] Hudson R.L., Moore M.H., Dworkin J.P., Martin M.P., Pozun Z.D. (2008). Amino Acids from Ion-Irradiated Nitrile-Containing Ices. Astrobiology.

[85] Pilling S., Duarte E.S., da Silveira E.F., Balanzat E., Rothard H., Domaracka A., Boduch P. (2010). Radiolysis of ammonia-containing ices by energetic, heavy, and highly charged ions inside dense astrophysical environments. Astron. Astrophys..

[86] Holtom P.D., Bennett C.J., Osamura Y., Mason N.J., Kaiser R.I. (2005). A combined experimental and theoretical study on the formation of the amino acid glycine (NH_2_CH_2_COOH) and its isomer (CH_3_NHCOOH) in extraterrestrial ices. Astrophys. J..

[87] Lafosse A., Bertin M., Domaracka A., Pliszka D., Illenberger E., Azria R. (2006). Reactivity induced at 25 K by low-energy electron irradiation of condensed NH_3_-CH_3_COOD (1:1) mixture. Phys. Chem. Chem. Phys..

[88] Esmaili S., Bass A.D., Cloutier P., Sanche L., Huels M.A. (2018). Glycine formation in CO_2_:CH_4_:NH_3_ ices induced by 0–70 eV electrons. J. Chem. Phys..

[89] Gerakines P.A., Moore M.H., Hudson R.L. (2004). Ultraviolet photolysis and proton irradiation of astrophysical ice analogs containing hydrogen cyanide. Icarus.

[90] Kanuchova Z., Urso R.G., Baratta G.A., Brucato J.R., Palumbo M.E., Strazzulla G. (2016). Synthesis of formamide and isocyanic acid after ion irradiation of frozen gas mixtures. Astron. Astrophys..

[91] Jones B.M., Bennett C.J., Kaiser R.I. (2011). Mechanistical Studies on the Production of Formamide (H_2_NCHO) within Interstellar Ice Analogs. Astrophys. J..

[92] Frigge R., Zhu C., Turner A.M., Abplanalp M.J., Bergantini A., Sun B.J., Chen Y.L., Chang A.H.H., Kaiser R.I. (2018). A Vacuum Ultraviolet Photoionization Study on the Formation of N-methyl Formamide (HCONHCH_3_) in Deep Space: A Potential Interstellar Molecule with a Peptide Bond. Astrophys. J..

[93] Bennett C.J., Kaiser R.I. (2007). On the formation of glycolaldehyde (HCOCH_2_OH) and methyl formate (HCOOCH_3_) in interstellar ice analogs. Astrophys. J..

[94] Maity S., Kaiser R.I., Jones B.M. (2015). Formation of complex organic molecules in methanol and methanol-carbon monoxide ices exposed to ionizing radiation—A combined FTIR and reflectron time-of-flight mass spectrometry study. Phys. Chem. Chem. Phys..

[95] Muñoz Caro G.M., Meierhenrich U.J., Schutte W.A., Barbier B., Segovia A.A., Rosenbauer H., Thiemann W.H.P., Brack A., Greenberg J.M. (2002). Amino acids from ultraviolet irradiation of interstellar ice analogues. Nature.

[96] Bernstein M.P., Dworkin J.P., Sandford S.A., Cooper G.W., Allamandola L.J. (2002). Racemic amino acids from the ultraviolet photolysis of interstellar ice analogues. Nature.

[97] Elsila J.E., Dworkin J.P., Bernstein M.P., Martin M.P., Sandford S.A. (2007). Mechanisms of amino acid formation in interstellar ice analogs. Astrophys. J..

[98] Pilling S., Andrade D.P.P., Neto A.C., Rittner R., de Brito A.N. (2009). DNA Nucleobase Synthesis at Titan Atmosphere Analog by Soft X-rays. J. Phys. Chem. A.

[99] Meinert C., Myrgorodska I., de Marcellus P., Buhse T., Nahon L., Hoffmann S.V., d’Hendecourt L.L., Meierhenrich U.J. (2016). Ribose and related sugars from ultraviolet irradiation of interstellar ice analogs. Science.

[100] Nuevo M., Cooper G., Sandford S.A. (2018). Deoxyribose and deoxysugar derivatives from photoprocessed astrophysical ice analogues and comparison to meteorites. Nat. Commun..

[101] Oba Y., Takano Y., Naraoka H., Watanabe N., Kouchi A. (2019). Nucleobase synthesis in interstellar ices. Nat. Commun..

[102] Chen Y.J., Ciaravella A., Muñoz Caro G.M., Cecchi-Pestellini C., Jiménez-Escobar A., Juang K.J., Yih T.S. (2013). Soft x-ray irradiation of methanol ice: Formation of products as a function of photon energy. Astrophys. J..

[103] Henderson B.L., Gudipati M.S. (2015). Direct detection of complex organic products in ultraviolet (lyα) and electron-irradiated astrophysical and cometary ice analogs using two-step laser ablation and ionization mass spectrometry. Astrophys. J..

[104] Ciaravella A., Jimenez-Escobar A., Cecchi-Pestellini C., Huang C.H., Sie N.E., Caro G.M.M., Chen Y.J. (2019). Synthesis of Complex Organic Molecules in Soft X-Ray Irradiated Ices. Astrophys. J..

[105] Martin-Domenech R., Oberg K., Rajappan M. (2020). Formation of NH_2_CHO and CH_3_CHO upon UV Photoprocessing of Interstellar Ice Analogs. Astrophys. J..

[106] Ciaravella A., Muñoz Caro G.M., Jiménez-Escobar A., Cecchi-Pestellini C., Hsiao L.-C., Huang C.-H., Chen Y.-J. (2020). X-ray processing of a realistic ice mantle can explain the gas abundances in protoplanetary disks. Proc. Natl. Acad. Sci. USA.

[107] Fraser H.J., Collings M.P., McCoustra M.R.S. (2002). Laboratory surface astrophysics experiment. Rev. Sci. Instrum..

[108] Teolis B.D., Loeffler M.J., Raut U., Fama A., Baragiola R.A. (2007). Infrared reflectance spectroscopy on thin films: Interference effects. Icarus.

[109] Coustenis A., Hirtzig M. (2009). Cassini-Huygens results on Titan’s surface. Res. Astron. Astrophys..

[110] Krüger H., Stephan T., Engrand C., Briois C., Siljestrom S., Merouane S., Baklouti D., Fischer H., Fray N., Hornung K. (2015). Cosima-Rosetta calibration for in situ characterization of 67P/Churyumov-Gerasimenko cometary inorganic compounds. Planet. Space Sci..

[111] Noble J.A., Theule P., Duvernay F., Danger G., Chiavassa T., Ghesquiere P., Mineva T., Talbi D. (2014). Kinetics of the NH_3_ and CO2 solid-state reaction at low temperature. Phys. Chem. Chem. Phys..

[112] Duvernay F., Dufauret V., Danger G., Theule P., Borget F., Chiavassa T. (2010). Chiral molecule formation in interstellar ice analogs: Alpha-aminoethanol NH_2_CH(CH_3_)OH. Astron. Astrophys..

[113] Abplanalp M.J., Forstel M., Kaiser R.I. (2016). Exploiting single photon vacuum ultraviolet photoionization to unravel the synthesis of complex organic molecules in interstellar ices. Chem. Phys. Lett..

[114] Nuevo M., Milam S.N., Sandford S.A. (2012). Nucleobases and Prebiotic Molecules in Organic Residues Produced from the Ultraviolet Photo-Irradiation of Pyrimidine in NH_3_ and H_2_O+NH_3_ Ices. Astrobiology.

[115] Karas M., Hillenkamp F. (1988). Laser desorption ionization of proteins with molecular masses exceeding 10,000 daltons. Anal. Chem..

[116] Beavis R.C., Chait B.T., Fales H.M. (1989). Cinnamic acid derivatives as matrices for ultraviolet laser desorption mass spectrometry of proteins. Rapid Commun. Mass Spectrom..

[117] Merchant M., Weinberger S.R. (2000). Recent advancements in surface-enhanced laser desorption/ionization-time of flight-mass spectrometry. Electrophoresis.

[118] Paardekooper D.M., Bossa J.B., Isokoski K., Linnartz H. (2014). Laser desorption time-of-flight mass spectrometry of ultraviolet photo-processed ices. Rev. Sci. Instrum..

[119] Yang R., Gudipati M.S. (2014). Novel two-step laser ablation and ionization mass spectrometry (2S-LAIMS) of actor-spectator ice layers: Probing chemical composition of D_2_O ice beneath a H_2_O ice layer. J. Chem. Phys..

[120] Strupat K., Karas M., Hillenkamp F. (1991). 2,5-Dihydroxybenzoic acid: A new matrix for laser desorption—Ionization mass spectrometry. Int. J. Mass Spectrom. Ion Process..

[121] Dreisewerd K. (2003). The Desorption Process in MALDI. Chem. Rev..

[122] Berkenkamp S., Menzel C., Karas M., Hillenkamp F. (1997). Performance of Infrared Matrix-assisted Laser Desorption/Ionization Mass Spectrometry with Lasers Emitting in the 3 μm Wavelength Range. Rapid Commun. Mass Spectrom..

[123] Caldwell K.L., Murray K.K. (1998). Mid-infrared matrix assisted laser desorption ionization with a water/glycerol matrix. Appl. Surf. Sci..

[124] Pirkl A., Soltwisch J., Draude F., Dreisewerd K. (2012). Infrared Matrix-Assisted Laser Desorption/Ionization Orthogonal-Time-of-Flight Mass Spectrometry Employing a Cooling Stage and Water Ice as a Matrix. Anal. Chem..

[125] Focsa C., Mihesan C., Ziskind M., Chazallon B., Therssen E., Desgroux P., Destombes J.L. (2006). Wavelength-selective vibrationally excited photodesorption with tunable IR sources. J. Phys. Condens. Matter.

[126] Maity S., Kaiser R.I., Jones B.M. (2014). Formation of ketene (H_2_CCO) in interstellar analogous methane (CH_4_)-carbon monoxide (CO) ices: A combined FTIR and reflectron time-of-flight mass spectroscopic study. Astrophys. J..

[127] Zhang J., Han F., Pei L., Kong W., Li A. (2010). Far-Infrared Spectroscopy of Cationic Polycyclic Aromatic Hydrocarbons: Zero Kinetic Energy Photoelectron Spectroscopy of Pentacene Vaporized from Laser Desorption. Astrophys. J..

[128] Hama T., Watanabe N., Kouchi A., Yokoyama M. (2011). Spin Temperature of Water Molecules Desorbed from the Surfaces of Amorphous Solid Water, Vapor-Deposited and Produced from Photolysis of a Ch_4_/O_2_ Solid Mixture. Astrophys. J. Lett..

[129] Gavilan L., Lemaire J.L., Vidali G., Sabri T., Jaeger C. (2014). The Formation of Molecular Hydrogen on Silicate Dust Analogs: The Rotational Distribution. Astrophys. J..

[130] Karas M., Bachmann D., Hillenkamp F. (1985). Influence of the wavelength in high-irradiance ultraviolet laser desorption mass spectrometry of organic molecules. Anal. Chem..

[131] Clark A.E., Kaleta E.J., Arora A., Wolk D.M. (2013). Matrix-Assisted Laser Desorption Ionization-Time of Flight Mass Spectrometry: A Fundamental Shift in the Routine Practice of Clinical Microbiology. Clin. Microbiol. Rev..

[132] Knochenmuss R. (2006). Ion formation mechanisms in UV-MALDI. Analyst.

[133] Trimpin S., Wang B., Inutan E.D., Li J., Lietz C.B., Harron A., Pagnotti V.S., Sardelis D., McEwen C.N. (2012). A Mechanism for Ionization of Nonvolatile Compounds in Mass Spectrometry: Considerations from MALDI and Inlet Ionization. J. Am. Soc. Mass Spectrom..

[134] Niu S., Zhang W., Chait B.T. (1998). Direct comparison of infrared and ultraviolet wavelength matrix-assisted laser desorption/ionization mass spectrometry of proteins. J. Am. Soc. Mass Spectrom..

[135] Luo Y., Li T., Yu F., Kramer T., Cristea I.M. (2010). Resolving the composition of protein complexes using a MALDI LTQ Orbitrap. J. Am. Soc. Mass Spectrom..

[136] Gudipati M.S., Yang R. (2012). In-situ probing of radiation-induced processing of organics in astrophysical ice analogs—Novel laser desorption laser ionization time-of-flight mass spectroscopic studies. Astrophys. J. Lett..

[137] Xian F., Hendrickson C.L., Marshall A.G. (2012). High Resolution Mass Spectrometry. Anal. Chem..

[138] Zubarev R.A., Makarov A. (2013). Orbitrap Mass Spectrometry. Anal. Chem..

[139] Arevalo Jr R., Selliez L., Briois C., Carrasco N., Thirkell L., Cherville B., Colin F., Gaubicher B., Farcy B., Li X. (2018). An Orbitrap-based laser desorption/ablation mass spectrometer designed for spaceflight. Rapid Commun. Mass Spectrom..

[140] Eliuk S., Makarov A. (2015). Evolution of Orbitrap Mass Spectrometry Instrumentation. Annu. Rev. Anal. Chem..

[141] Quack M., Merkt F.E. (2011). Handbook of High-Resolution Spectroscopy.

[142] Herman M., Georges R., Hepp M., Hurtmans D. (2000). High resolution Fourier transform spectroscopy of jet-cooled molecules. Int. Rev. Phys. Chem..

[143] Potapov A., Asselin P. (2014). High-resolution jet spectroscopy of weakly bound binary complexes involving water. Int. Rev. Phys. Chem..

[144] Grabow J.U. (2013). Fourier Transform Microwave Spectroscopy: Handedness Caught by Rotational Coherence. Angew. Chem. Int. Ed..

[145] Patterson D., Schnell M., Doyle J.M. (2013). Enantiomer-specific detection of chiral molecules via microwave spectroscopy. Nature.

[146] Yocum K.M., Smith H.H., Todd E.W., Mora L., Gerakines P.A., Milam S.N., Weaver S.L.W. (2019). Millimeter/Submillimeter Spectroscopic Detection of Desorbed Ices: A New Technique in Laboratory Astrochemistry. J. Phys. Chem. A.

[147] Harada K., Tanaka K., Tanaka T., Nanbu S., Aoyagi M. (2002). Millimeter-wave spectroscopy of the internal-rotation band of the He-HCN complex and the intermolecular potential energy surface. J. Chem. Phys..

[148] Potapov A.V., Surin L.A., Schlemmer S., Giesen T.F. (2011). Submillimeter-wave spectroscopy of the K = 2−1 subband of the Ne-CO complex. J. Mol. Spectrosc..

[149] Theulé P., Endres C., Hermanns M., Zingsheim O., Bossa J.B., Potapov A. (2020). High-Resolution Gas Phase Spectroscopy of Molecules Desorbed from an Ice Surface: A Proof-of-Principle Study. ACS Earth Space Chem..

[150] Brown G.G., Dian B.C., Douglass K.O., Geyer S.M., Shipman S.T., Pate B.H. (2008). A broadband Fourier transform microwave spectrometer based on chirped pulse excitation. Rev. Sci. Instrum..

[151] Jahn M.K., Dewald D.A., Wachsmuth D., Grabow J.U., Mehrotra S.C. (2012). Rapid capture of large amplitude motions in 2,6-difluorophenol: High-resolution fast-passage FT-MW technique. J. Mol. Spectrosc..

[152] Perez C., Lobsiger S., Seifert N.A., Zaleski D.P., Temelso B., Shields G.C., Kisiel Z., Pate B.H. (2013). Broadband Fourier transform rotational spectroscopy for structure determination: The water heptamer. Chem. Phys. Lett..

[153] Medcraft C., Wolf R., Schnell M. (2014). High-Resolution Spectroscopy of the Chiral Metal Complex [CpRe(CH_3_)(CO)(NO)]: A Potential Candidate for Probing Parity Violation. Angew. Chem. Int. Ed..

[154] Hermanns M., Wehres N., Lewen F., Muller H.S.P., Schlemmer S. (2019). Rotational spectroscopy of the two higher energy conformers of 2-cyanobutane. J. Mol. Spectrosc..

[155] Dian B.C., Brown G.G., Douglass K.O., Pate B.H. (2008). Measuring picosecond isomerization kinetics via broadband microwave spectroscopy. Science.

[156] Abeysekera C., Zack L.N., Park G.B., Joalland B., Oldham J.M., Prozument K., Ariyasingha N.M., Sims I.R., Field R.W., Suits A.G. (2014). A chirped-pulse Fourier-transform microwave/pulsed uniform flow spectrometer. II. Performance and applications for reaction dynamics. J. Chem. Phys..

[157] Prozument K., Park G.B., Shaver R.G., Vasiliou A.K., Oldham J.M., David D.E., Muenter J.S., Stanton J.F., Suits A.G., Ellison G.B. (2014). Chirped-pulse millimeter-wave spectroscopy for dynamics and kinetics studies of pyrolysis reactions. Phys. Chem. Chem. Phys..

[158] Abeysekera C., Joalland B., Ariyasingha N., Zack L.N., Sims I.R., Field R.W., Suits A.G. (2015). Product Branching in the Low Temperature Reaction of CN with Propyne by Chirped-Pulse Microwave Spectroscopy in a Uniform Supersonic Flow. J. Phys. Chem. Lett..

